# Morphometry and Morphology of Vascular Foramen on the Trochlear Groove of the Distal Femur and Its Implications

**DOI:** 10.7759/cureus.18954

**Published:** 2021-10-21

**Authors:** Rajani Singh

**Affiliations:** 1 Anatomy, Uttar Pradesh University of Medical Sciences, Saifai, IND

**Keywords:** trochlear groove, nutrient foramen, vascular foramen, distal femur, trochlear foramen

## Abstract

Background and objective

Nutrient foramina in supracondylar, medial condyle, lateral condyle, and intercondylar fossa have been described in the literature. The author of the present study observed a vascular foramen on the trochlear groove, which has not been previously reported in the literature. The aim of the study was to elaborate on the morphology, morphometry, and associated clinical implications of this foramen.

Materials and methods

Forty-five unpaired femora of unknown sex available in the Department of Anatomy at the Uttar Pradesh University of Medical Science (UPUMS), Saifai, India were observed to examine the incidence, shape, size, and location of the foramen on the trochlear groove of the distal femur. The incidence and shape were observed and its location in relation to the upper and lower articulating margin of the patella was recorded using a divider and scale.

Results

The trochlear foramen was observed in 25 femora, accounting for 55.6% of the total. The average diameter of the foramen was 4 mm. The average distance of the foramen from the upper patellar articulating surface was 2.4 cm and its distance from the lower margin was 0.7 cm. All the foramina were round in shape. The largest number of foramina were located in the midline, and seven foramina were located to the left of the midline of the trochlear groove.

Conclusions

The incidence of the trochlear foramen was 55.6%, meaning that more than half of the population possess this foramen on the trochlear groove. The maximum number of these foramen, amounting to 64%, were found in the midline of the trochlear groove. The morphologic and topographic knowledge of the trochlear foramen is essential for orthopedic surgeons during operative procedures in the region of the distal femur.

## Introduction

The bone is a highly vascular structure and a typical long bone is irrigated by four arterial systems, namely epiphyseal, diaphyseal, metaphyseal, and periosteal arteries. These are known as nutrient arteries or vascular channels. The femur is the long bone of the thigh and its distal end is supplied by the branches of the descending genicular, lateral circumflex, and fouth perforating arteries.

The arteries that supply this long bone enter into it via numerous foramina known as nutrient/vascular foramina. The nutrient artery enters the bone through the vascular foramen, passes through the nutrient canal, and finally opens into the marrow cavity. The nutrient artery irrigates the outer two-thirds of the cortex of the bone, and bone remodeling along with its regeneration depends chiefly on arterial supply [1]. An awareness of the detailed and precise location of the vascular foramen is essential for surgeons to prevent intraoperative damage to the artery passing through it [2]. Moreover, knowledge of the location of vascular foramina is essential to differentiate them from the fracture line [3]. The vascular foramen on the upper end, shaft, and lower end of the femur has been described by various researchers. But the author of this study observed a trochlear foramen on the trochlear groove of the distal femur, which has not been previously reported. The aim of the study was to elaborate on the incidence, shape, morphometry, and topography of this foramen and to describe the associated clinical significance.

## Materials and methods

Twenty-one left and 24 right femora, constituting 45 femora in total, of unknown sex obtained from the osteology lab of the Uttar Pradesh University of Medical Science (UPUMS) were observed to examine the incidence, shape, size, and location of vascular foramen present on the patellar surface of the femur also known as the trochlear groove. As the foramen was found on the trochlear groove of the distal femur, it is named ‘trochlear foramen’. Only those femora with their lower-end intact were considered for the study, and those with damaged lower ends were excluded.

**Figure 1:**
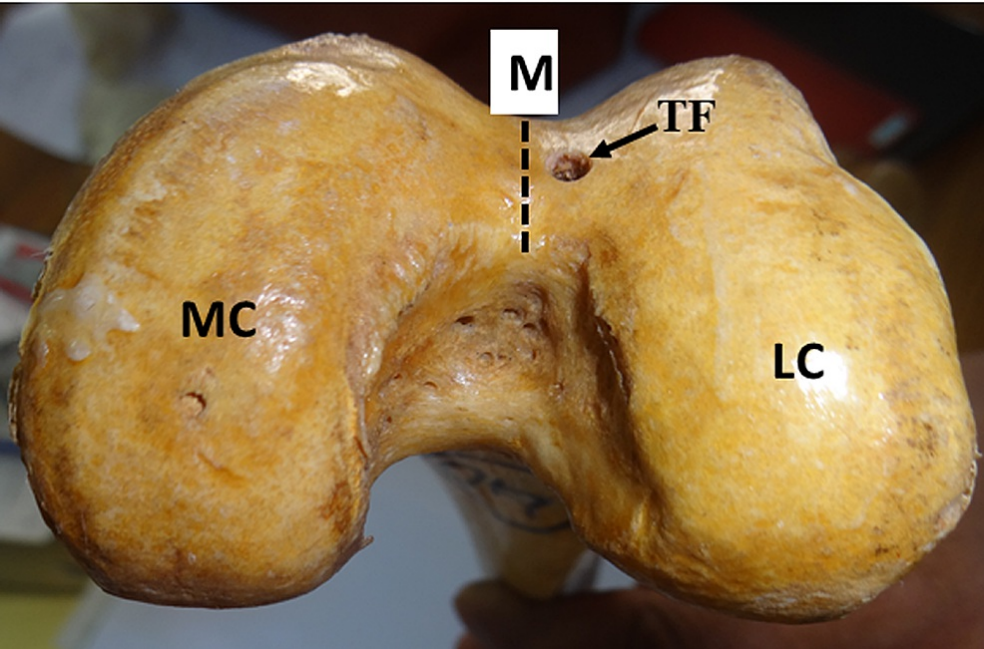
Trochlear foramen on the left of the midline on the trochlear groove of the distal femur TF: trochlear foramen; M: midline, MC: medial condyle; LC: lateral condyle

As the foramina were observed at variant locations, in order to assist orthopedic surgeons performing surgery around the distal femur, these foramina were classified into four groups: 1. Those on the left of the midline on the trochlear groove (Figure 1).

**Figure 2:**
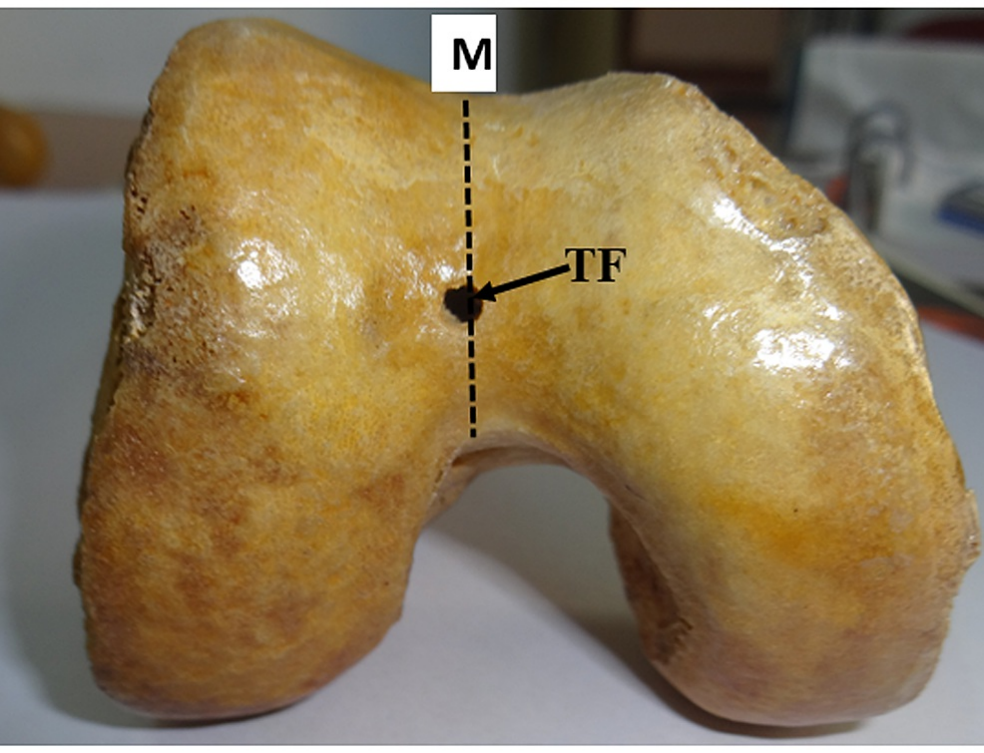
Trochlear foramen in the midline on the trochlear groove of the distal femur TF: trochlear foramen; M: midline

**Figure 3:**
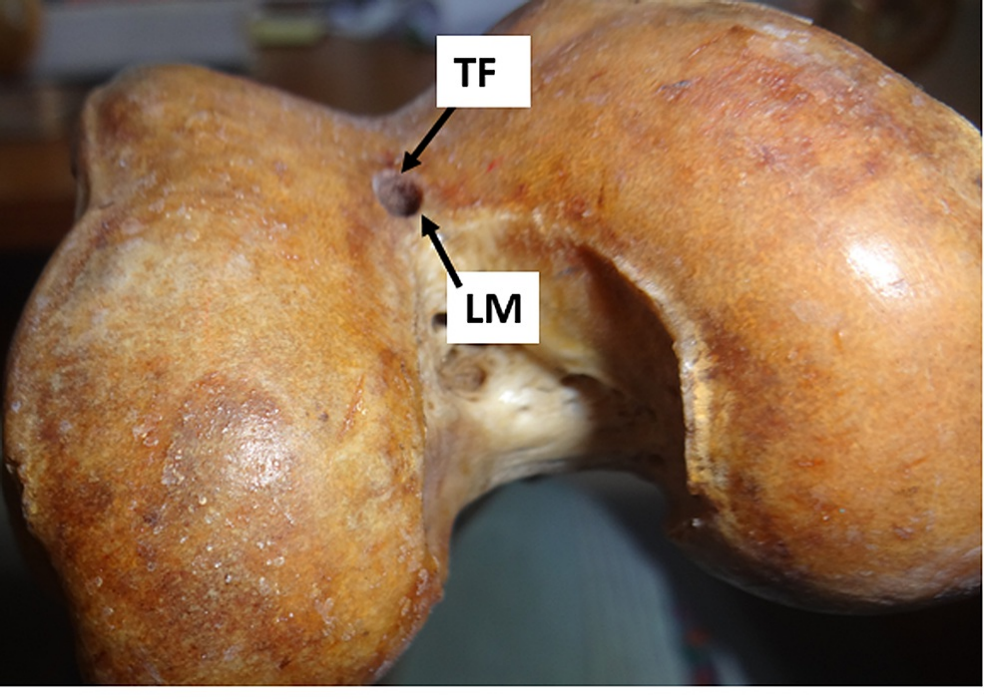
Trochlear foramen in the midline at the lower margin of the trochlear groove TF: trochlear foramen; M: midline

 2. Those in the midline at a variable distance from the upper and lower margins of the trochlear groove (Figure 2). 

3. Those in the midline at the lower margin of the trochlear groove (Figure 3).

4. Those on the right of the midline on the trochlear groove.

The shapes and sizes were observed and measurements of diameter and distance from the upper and lower margins of the trochlear groove were taken by a divider and scale. The percentage of incidence, the range, and the means of diameters and distances of foramina from the upper and lower margins of the trochlear groove were computed.

The literature was surveyed using different resources like PubMed, Scielo, ResearchGate, and Google, and various terms related to this foramen like "vascular foramen, nutrient foramen, the prevalence of vascular foramen, the significance of vascular foramen", etc. were used to elicit the clinical significance of this foramen.

## Results

All the foramina were round in shape. A single foramen was observed in all the 25 femora (55.6%). Total femora observed were 45, out of which 25 were detected to have a single trochlear foramen. Out of the 25 femora, 12 were right femora and 13 were left. Thus, this single trochlear foramen was detected at a rate of 48% in the right femora and 52% in the left femora out of a total of 25 femora.

The diameter of the foramen ranged between 3-5 mm. The average diameter of the foramen was 4 mm. The distance of the foramen from the upper margin of the trochlear groove ranged between 2-3 cm and that from the lower margin of the trochlear groove ranged between 0.5-1 cm. The average distance of the foramen from the upper margin of the trochlear groove of the femur was 2.4 cm and its distance from the lower margin was 0.7 cm.

The maximum number of trochlear foramina, amounting to 18 (72%), were located in the midline, out of which two were lying on the lower margin of the trochlear groove and the remaining 16 were in the midline at variable distances from the upper and lower margins of the trochlear groove as illustrated above. Seven (28%) foramina were located to the left of the midline. No foramen was found to be located to the right of the midline.

## Discussion

Vascular foramina in supracondylar, medial condyle, lateral condyle, and intercondylar fossa of the distal femur have been widely delineated in the literature. However, to the best of the author's knowledge, there is a paucity of literature describing the incidence, shape, size, and topography of foramen located on the trochlear groove of the distal femur. 

In this study, trochlear foramen was observed individually on 25 femora out of a total of 45 femora, constituting a rate of incidence of 55.6%. No literature is currently available describing the incidence of this foramen for comparison.

Anatomical characteristics of the vascular foramen like its number, position, size, shape, and direction are important parameters to be taken into account in orthopedic surgeries including bone grafting and fracture repair. These factors are essential for deciding the prognosis after a fracture because recovery depends on blood supply, which in turn depends on the aforementioned factors [4].

The number of vascular foramina is important because the higher the number of foramina the more vascular networking will be present in the area. A large number of foramina is advantageous as the area will be richly supplied and hence healing will be fast. But in our case, a single trochlear foramen was found in all the femora observed. This could be a causative cofactor for osteochondrosis dissecans [5]. Hence, this area could be the most frequent site for osteochondrosis dissecans in the knee joint.

The size of the foramen is also crucial as large nutrient vessels carrying more amount of blood reach the site, and so healing of fractures is rapid; however, the disadvantage is that if the vessel passing through such foramen is injured due to any reason, bleeding will be severe. In our case, the average size of the foramen was 4 mm and some foramina were even as large as 5 mm in diameter, which is quite large in size. Hence, bone flap harvest can be carried out with the least complications. In addition to this large-sized trochlear foramen as seen in our study, furnishing sufficient periosteal blood supply to bone allows adequate corticocancellous bone flap harvest without jeopardizing the nutrition [6]. The high osteogenic potential of the cambium layer and larger size of vascular foramen make this area an ideal one for preventing bony non-union.

Osteotomy of the distal femur is a common procedure for realigning a varus or valgus leg alignment. But this procedure is usually followed by substantial bleeding. So, the periosteal feeders or larger artery near the bone cuts should be protected during the procedure [7] since, as in our case, the vascular foramen transmits through quite a large artery (artery with a 4-mm diameter).

The distal femur is pivotal from the anatomical, functional, and clinical points of view as it is exposed to serious morbidity due to its position, structure, and weight‑bearing function [8].

Osteoarthritis and fractures very frequently involve the knee joint and lower end of the femur and these are commonly used for vascularised bone grafts. Comprehension of the vascular anatomy of the distal femur and knee joint is very crucial as surgical interventions in these areas may cause ischemic changes, which can be hazardous for the patient [9,10]. Knowing the vascular pattern around the knee from the perspective of osteotomies is critical to prevent potential complications. Also, awareness of the arterial anatomy of bones employed for harvesting vascularized bone flaps is very essential as the localization of vascular pedicles of larger size will ensure the vitality of graft as well as the donor site and also avoid osteonecrosis.

The fracture of long bones including the distal femur causes rupture of arteries passing through vascular foramen culminating in local bleeding. Thus, knowledge of vascular foramina morphometry is of paramount importance in some orthopedic surgical procedures, such as joint replacement therapy, fracture repair, bone grafts, vascularized bone microsurgery, and also in medico-legal cases [11]. In free vascular bone grafting, the blood supply by the nutrient artery is very important and must be preserved to promote fracture healing.

In the lower limb, the lower end of the femur and the upper end of the tibia are the growing ends. The nutrient arteries along with veins pass through vascular foramen. One of the factors responsible for delayed union or non-union of fracture is the lack of arterial supply. It is a known fact that the process of repair of a traumatic or surgically induced fracture takes place very slowly. Therefore, the morphological knowledge of vascular foramen is very essential for orthopedic surgeons carrying out an open reduction of a fracture to avoid injuring the nutrient artery and thus decreasing incidences of delayed/non-union of the fracture [12]. The external opening of the vascular foramen is located at a particular position in each bone. In our case, vascular foramen was found to be situated at three different locations; maximum foramen (16) in the midline, two at the lower articulating margin of the patellar surface in the midline, and seven to the left of the midline. No literature is available describing the location and position of this foramen for comparison. It is said that the vessels that occupy the vascular foramen are derived from those that initially invaded the ossifying cartilage, and hence the vascular foramen was at the site of the original center of ossification. Hughes observed that the variant foramina were common in the femur, rare in the radius, and very rare in other bones [13]. Variations in the direction of nutrient foramina have been observed in many tetrapods and there is some similarity in the foraminal pattern in mammals and birds [14].

The nutrient artery travels away from the growing end as the growing bone might pull and rupture the artery. So, the vascular foramina are directed away from the growing end. The position of vascular foramina is influenced by two factors; growth rates at two ends of the shaft and bone remodeling. Studies are to be carried out on growth rates and bone remodeling in the bones of different age groups. Moreover, fetal bones could also help in analyzing this concept. In the present study, these factors could not be analyzed as the study involves adult dry bones. In the present study, the maximum number of trochlear foramina, constituting 64%, were located in the midline of the trochlear groove, while 28% was located on the left condyle, a little left of the midline, and 8% at the lower margin of the trochlear groove. There is no literature describing the position of the trochlear foramen and hence a comparison is not possible.

Nutrient arteries irrigating long bones are vital during the active growth period in the embryo and fetus and at the early phases of ossification and so these arteries should be kept patent until the growth is completed, and even after the growth is complete. Therefore, these are directed away from the growing end [15]. However, the trochlear foramen in the present study was in the region of growth and so was directed anteroposteriorly and not upward or downward from the growing end. The pull of muscle attachments on the periosteum could also contribute to the anomalous direction of vascular foramina.

The foramen may be a potential area of weakness in some patients, and when under stress, either because of increased physical activity or the decreased quality of the bone, the foramen may lead to the development of fractures. The position of the fracture relative to the vascular foramen of the long bone and the patterns of edema are the key secondary signs in the diagnosis of this type of fracture. Thus, knowledge about the morphometry, morphology, and topography of the trochlear foramen in the trochlear groove of the distal femur is very essential for orthopedic surgeons.

## Conclusions

The incidence of the trochlear foramen is 55.6%, meaning that more than half of the population possesses this foramen on the trochlear groove. The maximum number of these foramen, amounting to 64%, were found in the midline of the trochlear groove. The morphologic, morphometric, and topographic knowledge of the trochlear foramen is very essential for orthopedic surgeons during bone graft preparation, tumor resections, traumas, congenital pseudoarthrosis, instrumentation and fixation, and in transplant techniques in orthopedics and other operative procedures in the region of the distal femur and knee to preserve the circulation. Moreover, information on the location of this foramen is of utmost importance for bone ossification, bone healing, and microvascular bone grafting.
